# A robust Pearson correlation test for a general point null using a surrogate bootstrap distribution

**DOI:** 10.1371/journal.pone.0216287

**Published:** 2019-05-16

**Authors:** Alan D. Hutson

**Affiliations:** Department of Biostatistics and Bioinformatics, Roswell Park Comprehensive Cancer Center, Buffalo, NY 14263 United States of America; Dartmouth College Geisel School of Medicine, UNITED STATES

## Abstract

In this note we present a robust bootstrap test with good Type I error control for testing the general hypothesis *H*_0_: *ρ* = *ρ*_0_. In order to carry out this test we use what is termed a *surrogate bootstrap distribution*. The test was inspired by the studentized permutation for testing *H*_0_: *ρ* = 0, which was proven to be exact in certain scenarios and asymptotically correct overall. We show that the bootstrap based test is robust to a variety of distributional scenarios in terms of proper Type I error control.

## Introduction

The term “correlation” was introduced by Galton as a synonym for regression and the term “coefficient of correlation” was first used by Edgeworth, who also derived the first sample estimator of linear association [1], [2]. Estimation and testing based on the correlation coefficient is one of the most used approaches towards examining the association between two continuous variables. Even though Edgeworth first derived the estimator of the sample correlation coefficient it was Karl Pearson who is credited with much of its development via his 1896 paper [3], and why we often refer to the “Pearson Correlation Coefficient” as given by the form

$$\rho =\frac{\mu_{XY}}{\sigma_{X}\sigma_{Y}},$$ (1)

where −1 < *ρ* < 1, *μ*_*XY*_ is the covariance between two random variables *X* and *Y*, *σ*_*X*_ is the standard deviation of *X* and *σ*_*Y*_ is the standard deviation of *Y*.

Throughout this note let (*X*_1_, *Y*_1_), (*X*_2_, *Y*_2_), ⋯, (*X*_*n*_, *Y*_*n*_) be *n* paired observations from a non-degenerate joint distribution *F*_*XY*_(*x*, *y*). The well-known sample estimator for *ρ* at (1) is given as

$$\hat{\rho }=\frac{\sum_{i=1}^{n}\left(X_{i}-\bar{X}\right)\left(Y_{i}-\bar{Y}\right)}{\sqrt{\sum_{i=1}^{n}{\left(X_{i}-\bar{X}\right)}^{2}}\sqrt{\sum_{i=1}^{n}{\left(Y_{i}-\bar{Y}\right)}^{2}}},$$ (2)

where $\bar{X}=\sum_{i=1}^{n}X_{i}/n$ and $\bar{Y}=\sum_{i=1}^{n}Y_{i}/n$. The estimator $\hat{\rho }$ is also well-known to be the maximum likelihood estimator for the bivariate normal correlation parameter.

The sampling distribution for $\hat{\rho }$ at (2) under bivariate normality was derived by R. A. Fisher [4]. For the case when *ρ* = 0, “Student” [5] surmised that $\sqrt{\left(n-2\right){\hat{\rho }}^{2}/\left(1-{\hat{\rho }}^{2}\right)}$ has a *t*-distribution with *n* − 2 degrees-of-freedom. The *t*-distribution based test, assuming bivariate normality, for testing the hypothesis *H*_0_: *ρ* = 0 versus *H*_1_: *ρ* ≠ 0 is generally what is found in most statistical software packages. For the more general bivariate normal case when *ρ* ≠ 0 the exact distribution for $\hat{\rho }$ at (2) is a bit more unwieldy [1]. Remarkably, Fisher discovered a large sample asymptotic normal variance stabilized approximation based on what is now known as Fisher’s z-transformation, which is given as

$$z=\frac{1}{2}\text{log}\frac{1+\hat{\rho }}{1-\hat{\rho }}.$$ (3)

This transformed variable tends to normality much faster than the classic distribution free based asymptotic normal approximation. The first order variance of *z* at (3) is 1/(*n* − 3). Various refinements of Fisher’s work have been carried out over the years in terms of both bias reduction and refined variance expressions [1]. For the more general test of *H*_0_: *ρ* = *ρ*_0_ (including *H*_0_: *ρ* = 0 versus one-sided alternatives) Fisher’z z-transformation approach, again under bivariate normality assumptions, is what most current statistical software packages employ.

Alternatively, the “distribution free” large sample approximation for the sampling distribution for $\hat{\rho }$ may be derived using the multivariate delta method as outlined in Serfling [6] page 126 and given by first noting that we can express the sample estimator as $\hat{\rho }=g\left(V\right)$, where

$$V=(\bar{X},\bar{Y},\frac{1}{n}\sum_{i=1}^{n}X_{i}^{2},\frac{1}{n}\sum_{i=1}^{n}Y_{i}^{2},\frac{1}{n}\sum_{i=1}^{n}X_{i}Y_{i}),$$ (4)

such that as per Serfling [6] we have

$$\hat{\rho }∼AN\left(\rho ,n^{-1}d\Sigma d^{\prime }\right).$$ (5)

For expression (5) **Σ** is the 5 × 5 variance-covariance matrix for the vector of the components of the summands of **V** given by $\left(X_{1},Y_{1},X_{1}^{2},Y_{1}^{2},X_{1}Y_{1}\right)$. The elements of **d**, which will play a key role in our application are given as

$$\begin{array}{cc} d_{1} & =\frac{\rho \mu_{X}}{\sigma_{X}^{2}}-\frac{\mu_{Y}}{\sigma_{X}\sigma_{Y}},d_{2}=\frac{\rho \mu_{Y}}{\sigma_{Y}^{2}}-\frac{\mu_{X}}{\sigma_{X}\sigma_{Y}},\\ & d_{3}=-\frac{\rho }{2\sigma_{X}^{2}},d_{4}=-\frac{\rho }{2\sigma_{Y}^{2}},d_{5}=\frac{1}{\sigma_{X}\sigma_{Y}}. \end{array}$$ (6)

More refined approximations for the sampling distribution of $\hat{\rho }$ via Edgeworth techniques for non-normal populations [7], [8].

There have been several investigations examining the robustness of the bivariate normality assumptions as it pertains to type I error control for testing *H*_0_: *ρ* = *ρ*_0_ using Fisher’s z-transformation or the more specific case of *H*_0_: *ρ* = 0 using Student’s *t* distribution. One of the earliest of these investigations is due to Pearson [9] who concluded that “the results suggest that the normal bivariate surface can be mutilated and distorted to a remarkable degree without affecting the frequency distribution of *r*”, which has since been disproved [7]. Havlicek and Peterson [10] also incorrectly concluded “that the Pearson r is insensitive to extreme violations of the basic assumptions of normality.” There has been a substantial number of simulation based studies, primarily in the psychology literature that note when tests about the correlation coefficient, assuming bivariate normality incorrectly, tend to fail [11]–[15].

We will repeat the simulation study of DiCiccio and Romano [16] and re-examine the Type I error control for testing *H*_0_: *ρ* = *ρ*_0_ based on Fisher’s z-transformation, the classic large sample approximation and our new bootstrap approach. To the best of our knowledge there does not seem to be an extensive simulation study of the straightforward asymptotic approximation above given by using the corresponding moment estimators in place of their population counterparts and setting *ρ* = *ρ*_0_ under the null. DiCiccio and Romano [16] do include results for the specific case for testing *H*_0_: *ρ* = 0.

As an alternative to either assuming bivariate normality or using a large sample asymptotic approximation one may consider a permutation test approach for the specific test *H*_0_: *ρ* = 0 [17]. The key sticking point that is often overlooked by practitioners is that the permutation test is only exact in terms of Type I error control when using the metric *ρ* to test *H*_0_: *F*_*XY*_ = *F*_*X*_ *F*_*Y*_. Only under specific distributional assumptions, e.g. bivariate normality or certain families of elliptical distributions, is the permutation test exact for testing *H*_0_: *ρ* = 0. Heuristic investigations for permutation testing about the correlation coefficient go back several decades [18]. Even recently, Tuǧran [19] make the same mistakes of the past when drawing conclusions regarding the permutation test’s applicability under normality versus non-normality.

It was only until very recently that DiCiccio and Romano [16] provided a careful theoretical treatment of the subject summarizing the methodologies and providing guidance for the most appropriate permutation approach for testing *H*_0_: *ρ* = 0. In their work, DiCiccio and Romano [16] provide a novel method of obtaining a permutation test that is exact when testing *H*_0_: *ρ* = 0 is equivalent to testing *H*_0_: *F*_*XY*_ = *F*_*X*_ *F*_*Y*_ and asymptotically controls the Type I error when testing *H*_0_: *ρ* = 0 is not equivalent to testing *H*_0_: *F*_*XY*_ = *F*_*X*_ *F*_*Y*_. The key to their approach is standardizing the test statistic using the large sample variance at (5) under *H*_0_: *ρ* = 0 such that as *n* → ∞ the quantiles of the studentized test statistic $\hat{\rho }/\sqrt{n^{-1}d\Sigma d^{\prime }}$ for the permutation distribution and the true sampling distribution “converge almost surely to the corresponding quantiles of the standard normal distribution.” In fact, the specific form of the standardization they utilize for testing *H*_0_: *ρ* = 0 can be traced back to Tschuprow [20].

As an overall strategy for testing *H*_0_: *ρ* = 0 the permutation testing approach that appears in DiCiccio and Romano [16] appears to be the most robust strategy to date in terms of controlling Type I error rates across a variety of distributional assumptions. In particular it is clear that the over-reliance on the bivariate normality assumption, when testing *H*_0_: *ρ* = 0, and utilized in many statistical software packages is not a particularly robust assumption. As noted by R. C. Geary [21]: “Normality is a myth; there never was, and never will be, a normal distribution.” One might extend this quote in an even stronger fashion relative to bivariate normality relative to its existence in real-life data analysis problems and testing about the correlation coefficient.

In this note we build upon the work of DiCiccio and Romano [16] by introducing a bootstrap-like resampling approach for testing the general null hypothesis *H*_0_: *ρ* = *ρ*_0_, which has virtually the same properties as the permutation approach for testing about *H*_0_: *ρ* = 0. The general difference between our approach and the approach of DiCiccio and Romano [16] is that the permutation testing approach is essentially a sampling without replacement method under the null hypothesis while our approach is a sampling with replacement approach under the null hypothesis approach. This subtle difference will allow us to develop a more general testing approach for *H*_0_: *ρ* = *ρ*_0_. Both approaches rely on a properly standardized test statistic based on (5) under *H*_0_: *ρ* = 0 such that the large sample Type I error control controlled exactly for certain scenarios and in general asymptotically. The test can also be inverted to provide precise confidence interval for *ρ* as an alternative to the more standard bootstrap confidence interval methods [22].

## Materials and methods

The main thrust of the bootstrap correlation test of *H*_0_: *ρ* = *ρ*_0_ is to generalize the approach of DiCiccio and Romano [16] by noting that we can approximate the distribution function of the sample correlation estimator $\hat{\rho }|H_{0}$ given *F*_*XY*_|*H*_0_ using a surrogate distribution function, which we will describe below. Bootstrap samples will be drawn from the surrogate distribution function *F*_*ST*_|*H*_0_ ≈ *F*_*XY*_|*H*_0_, which is described in detail below.

In terms ofthe preliminaries let (*X*_1_, *Y*_1_), (*X*_2_, *Y*_2_), ⋯, (*X*_*n*_, *Y*_*n*_) be an i.i.d. bivariate sample from an absolutely continuous distribution *F*_*XY*_ with marginal distributions denoted *F*_*X*_ and *F*_*Y*_. In terms of our application let the 2 × 2 positive definite correlation matrix for the standardized variables $\left(\frac{X_{1}-\mu_{X}}{\sigma_{X}},\frac{Y_{1}-\mu_{Y}}{\sigma_{Y}}\right)$ be given as as

$$\Gamma =(\begin{array}{cc} 1 & \rho \\ \rho & 1 \end{array}).$$ (7)

Next, represent the Cholesky decomposition of the *p* × *p* matrix **Γ** as

$$\Gamma =A^{\prime }A,$$ (8)

such that **A**′^-1^ is defined. The Cholesky decomposition is a key component of the bootstrap test that we propose given below.

Denote the *n* × 2 matrix of standardized observations as (**U**⋮**V**), where

$$U^{{}^{\prime }}=(\frac{X_{1}-\mu_{X}}{\sigma_{X}},\frac{X_{2}-\mu_{X}}{\sigma_{X}},\cdots ,\frac{X_{n}-\mu_{X}}{\sigma_{X}})$$ (9)

and

$$V^{{}^{\prime }}=(\frac{Y_{1}-\mu_{Y}}{\sigma_{Y}},\frac{Y_{2}-\mu_{Y}}{\sigma_{Y}},\cdots ,\frac{Y_{n}-\mu_{Y}}{\sigma_{Y}}),$$ (10)

respectively. Apply the transformation

$${\left(S\vdots T\right)}^{\prime }=A^{\prime -1}{\left(U\vdots V\right)}^{\prime },$$ (11)

where *A* is the decomposed matrix at (8), where more specifically stated

$$A^{\prime -1}=(\begin{array}{cc} 1 & 0\\ \rho & \sqrt{1-\rho^{2}} \end{array}),$$ (12)

such that we have transformed observations are given as *S*_*i*_ = *U*_*i*_ and $T_{i}=\rho U_{i}+\sqrt{1-\rho^{2}}V_{i}$, *i* = 1, 2, ⋯, *n*.

Next denote $\sigma_{\hat{\rho }\left(X,Y\right)}^{2}=n^{-1}d_{\text{XY}}\Sigma_{\text{XY}}d_{\text{XY}}^{\prime }$, where **Σ**_**XY**_ is the 5 × 5 variance-covariance matrix for the vector $\left(X_{1},Y_{1},X_{1}^{2},Y_{1}^{2},X_{1}Y_{1}\right)$ given earlier at (5) and **d**_**XY**_ is the 1 × 5 vector defined at (6). For the transformed variables similarly denote $\sigma_{\hat{\rho }\left(S,T\right)}^{2}=n^{-1}d_{\text{ST}}\Sigma_{\text{ST}}d_{\text{ST}}^{\prime }$, where **Σ**_**ST**_ is the 5 × 5 variance-covariance matrix for the vector $\left(S_{1},T_{1},S_{1}^{2},T_{1}^{2},S_{1}T_{1}\right)$ and the vector **d**_**ST**_ is defined similar to **d**_**XY**_ defined at (6) now using the moments based on *S* and *T* in place of *X* and *Y*.

Under *H*_0_: *ρ* = *ρ*_0_ the parameter *ρ* is “known”. The estimator for the variance of $\hat{\rho }\left(X,Y\right)|H_{0}={\hat{\rho }}_{0}\left(X,Y\right)$ will be denoted as

$$s_{{\hat{\rho }}_{0}\left(X,Y\right)}^{2}=n^{-1}{\hat{d}}_{\text{XY},H_{0}}{\hat{\Sigma }}_{\text{XY}}{\hat{d}}_{\text{XY},H_{0}}^{\prime },$$ (13)

where population moments are replaced with their corresponding sample moment counterparts throughout $d_{XY,H_{0}}$ and *Σ*_*XY*_, and *ρ* = *ρ*_0_. In a similar fashion denote the estimator for the variance of $\hat{\rho }\left(S,T\right)|H_{0}={\hat{\rho }}_{0}\left(S,T\right)$ as

$$s_{{\hat{\rho }}_{0}\left(S,T\right)}^{2}=n^{-1}{\hat{d}}_{\text{ST},H_{0}}{\hat{\Sigma }}_{\text{ST}}{\hat{d}}_{\text{ST},H_{0}}^{\prime }.$$ (14)

**Theorem 2.1** Under *H*_0_: *ρ* = *ρ*_0_ assume (*X*_1_, *Y*_1_), (*X*_2_, *Y*_2_), ⋯, (*X*_*n*_, *Y*_*n*_) are an i.i.d. bivariate sample from an absolutely continuous distribution *F*_*XY*_. Also, assume $E(X_{1}^{4})<\infty$, $E(Y_{1}^{4})<\infty$ and $E(X_{1}^{2}Y_{1}^{2})<\infty$. Then as *n* → ∞

$$\frac{{\hat{\rho }}_{0}\left(X,Y\right)-\rho_{0}}{s_{{\hat{\rho }}_{0}\left(X,Y\right)}}\to AN\left(0,1\right).$$ (15)

The result follows straightforward noting that the sample moments used to calculate the estimator $s_{{\hat{\rho }}_{0}\left(X,Y\right)}$ are all averages and as *n* → ∞ all converge to their constant population counterparts. Hence, under the conditions outlined above $s_{{\hat{\rho }}_{0}\left(X,Y\right)}\to \sigma_{{\hat{\rho }}_{0}\left(X,Y\right)}$ as *n* → ∞. Noting that as per Serfling [6] page 126 that $\frac{{\hat{\rho }}_{0}\left(X,Y\right)-\rho_{0}}{\sigma_{{\hat{\rho }}_{0}\left(X,Y\right)}}\to AN\left(0,1\right)$ as *n* → ∞ completes the argument using Slutsky’s Theorem with the conditions $E(Y_{1}^{4})<\infty$ and $E(X_{1}^{2}Y_{1}^{2})<\infty$.

**Theorem 2.2** Under *H*_0_: *ρ* = *ρ*_0_ assume (*S*_1_, *T*_1_), (*S*_2_, *T*_2_), ⋯, (*S*_*n*_, *T*_*n*_) defined at (11) are an i.i.d. bivariate sample from an absolutely continuous distribution *F*_*ST*_. Also, assume $E(S_{1}^{4})<\infty$, $E(T_{1}^{4})<\infty$, $E(S_{1}^{2}T_{1}^{2})<\infty$ and that *μ*_*X*_, *σ*_*X*_, *μ*_*Y*_ and *σ*_*Y*_ are known. Then as *n* → ∞

$$\begin{array}{c} \frac{{\hat{\rho }}_{0}\left(S,T\right)-\rho_{0}}{s_{{\hat{\rho }}_{0}\left(S,T\right)}}\to AN\left(0,1\right). \end{array}$$ (16)

The result follows using the same arguments as in Theorem 2.1 given the key feature that *μ*_*X*_, *σ*_*X*_, *μ*_*Y*_ and *σ*_*Y*_ are known quantities.

**Comment 2.1** Under Theorem 2.1 and Theorem 2.2. we see that both estimators ${\hat{\rho }}_{0}\left(X,Y\right)$ and ${\hat{\rho }}_{0}\left(S,T\right)$ have the same large sample distributions and their expectations are given as $E\left({\hat{\rho }}_{0}\left(X,Y\right)|H_{0}\right)=E\left({\hat{\rho }}_{0}\left(S,T\right)|H_{0}\right)=\rho_{0}$.

**Comment 2.2** Relative to Theorem 2.2 we can generate bootstrap samples denoted as the pairs $\left(S_{1}^{*},T_{1}^{*}\right),\left(S_{2}^{*},T_{2}^{*}\right),\cdots ,\left(S_{n}^{*},T_{n}^{*}\right)$ under the null hypothesis *H*_0_: *ρ* = *ρ*_0_ such that $E\left({\hat{\rho }}_{0}\left(S^{*},T^{*}\right)|H_{0}\right)\to \rho_{0}$. This is done by first drawing independent bootstrap samples from the marginal distribution functions ${\hat{F}}_{X}$ and ${\hat{F}}_{Y}$ and applying an empirical version of the transformation at (11), i.e. replace *μ*_*X*_, *σ*_*X*_, *μ*_*Y*_ and *σ*_*Y*_ with their respective sample counterparts. Essentially we are drawing nonparametric bootstrap samples from ${\hat{F}}_{ST}|H_{0}$ to estimate the bootstrap distribution of the sample variance for ${\hat{\rho }}_{0}\left(S,T\right)|H_{0}$ with the scale based on the underlying properties of the distribution *F*_*ST*_|*H*_0_. We can then rescale based on the sample variance for ${\hat{\rho }}_{0}\left(X,Y\right)|H_{0}$ in order to have an asymptotically valid testing procedure. More specifically we can follow the the steps below to test *H*_0_: *ρ* = *ρ*_0_ versus *H*_1_: *ρ* > *ρ*_0_ as an extension of the rescaling ideas put forward by DiCiccio and Romano [16] for testing the specific hypothesis *H*_0_: *ρ* = 0. Note also that the procedure we propose can be modified easily to handle the alternatives *H*_1_: *ρ* < *ρ*_0_ and *H*_1_: *ρ* ≠ *ρ*_0_.

Out testing algorithm now follows these steps:

1. Calculate the Pearson sample correlation ${\hat{\rho }}_{0}\left(X,Y\right)$ (the subscript is added for notational convenience), which has the same functional form as $\hat{\rho }$ ($={\hat{\rho }}_{0}\left(X,Y\right)$) given at (2), and its corresponding variance estimate under *H*_0_, $s_{{\hat{\rho }}_{0}\left(X,Y\right)}^{2}$ given at (13).
2. Draw samples of size *n* independently and with replacement from each marginal distribution ${\hat{F}}_{X}$ and ${\hat{F}}_{Y}$ and denote the pairs of independently drawn observations as $\left(X_{1}^{*},Y_{1}^{*}\right),\left(X_{2}^{*},Y_{2}^{*}\right),\cdots ,\left(X_{n}^{*},Y_{n}^{*}\right)$, where ${\hat{F}}_{X}\left(x\right)=\sum_{i=1}^{n}I_{\left(x_{i}\leq x\right)}/n$ and ${\hat{F}}_{Y}\left(y\right)=\sum_{i=1}^{n}I_{\left(y_{i}\leq y\right)}/n$, respectively.
3. Standardize each observation using the observed sample means and sample standard deviations as $\left(\frac{X_{1}^{*}-\bar{X}}{s_{X}},\frac{Y_{1}^{*}-\bar{Y}}{s_{Y}}\right),\left(\frac{X_{2}^{*}-\bar{X}}{s_{X}},\frac{Y_{2}^{*}-\bar{Y}}{s_{Y}}\right),\cdots ,\left(\frac{X_{n}^{*}-\bar{X}}{s_{X}},\frac{Y_{n}^{*}-\bar{Y}}{s_{Y}}\right)$.
4. Denote the *n* × 2 matrix of standardized bootstrap observations as (U*⋮V*), where $U^{{*}^{\prime }}=(\frac{X_{1}^{*}-\bar{X}}{s_{X}},\frac{X_{2}^{*}-\bar{X}}{s_{X}},\cdots ,\frac{X_{n}^{*}-\bar{X}}{s_{X}})$ and $V^{{*}^{\prime }}=(\frac{Y_{1}^{*}-\bar{Y}}{s_{Y}},\frac{Y_{2}^{*}-\bar{Y}}{s_{Y}},\cdots ,\frac{Y_{n}^{*}-\bar{Y}}{s_{Y}})$, respectively.
5. Apply the transformation $A_{0}^{\prime -1}{\left(U^{*}\vdots V^{*}\right)}^{\prime }$, where *A*_0_ is the decomposed matrix at (8) replacing *ρ* with *ρ*_0_, where specifically stated
  $$A_{0}^{\prime -1}=(\begin{array}{cc} 1 & 0\\ \rho_{0} & \sqrt{1-\rho_{0}^{2}} \end{array}),$$ (17)
  such that we have correlated bootstrap resampled paired values are given as $S_{i}^{*}=U_{i}^{*}$ and $T_{i}^{*}=\rho_{0}U_{i}^{*}+\sqrt{1-\rho_{0}^{2}}V_{i}^{*}$, *i* = 1, 2, ⋯, *n*.
6. Calculate ${\hat{\rho }}_{0}^{*}\left(S^{*},T^{*}\right)$ using the standard sample correlation formula (2) and the corresponding variance estimate $s_{{\hat{\rho }}_{0}^{*}\left(S,T\right)}^{2}$ at (14) under *H*_0_ using the correlated values $\left(S_{i}^{*},T_{i}^{*}\right)$, *i* = 1, 2, ⋯, *n*, to generate an empirical bootstrap null distribution of observations for a single bootstrap replicate.
7. Calculate the bootstrap rescaled and bias corrected bootstrap correlation estimate under *H*_0_ as ${\hat{\rho }}^{**}=\rho_{0}-\frac{s_{{\hat{\rho }}_{0}\left(X,Y\right)}}{s_{\hat{\rho_{0}^{*}\left(S,T\right)}}}\left({\hat{\rho }}^{*}\left(S^{*},T^{*}\right)-\rho_{0}\right)$.
8. Repeat steps 2-7 *B* times (usually *B* = 500 or *B* = 1000 is sufficient).
9. Calculate the bootstrap estimated p-value as $p=\frac{\sum_{j=1}^{B}I_{\left({\hat{\rho }}_{j}^{**}>\hat{\rho }\left(X,Y\right)\right)}}{B}$, where *I* denotes the standard indicator function.

An outline for the large sample validity of the test can be examined via Edgeworth expansion techniques [1], for an overview of Edgeworth expansion methods. Towards this end note that under *H*_0_: *ρ* = *ρ*_0_ we have

$$\begin{array}{ccc} P(\frac{{\hat{\rho }}_{0}\left(S,T\right)-\rho_{0}}{s_{{\hat{\rho }}_{0}\left(S,T\right)}}\leq c|F_{ST},H_{0}) & = & \Phi \left(c\right)+\frac{p_{1}\left(c|F_{ST},H_{0}\right)}{\sqrt{n}}\phi \left(c\right)\\ & + & \frac{p_{2}\left(c|F_{ST},H_{0}\right)}{n}\phi \left(c\right)+o_{p}(\frac{1}{n}) \end{array}$$ (18)

and

$$\begin{array}{ccc} P(\frac{{\hat{\rho }}_{0}^{*}\left(S,T\right)-\rho_{0}}{s_{{\hat{\rho }}_{0}^{*}\left(S,T\right)}}\leq c|{\hat{F}}_{ST},H_{0}) & = & \Phi \left(c\right)+\frac{p_{1}\left(c|{\hat{F}}_{ST}^{*},H_{0}\right)}{\sqrt{n}}\phi \left(c\right)\\ & + & \frac{p_{2}\left(c|{\hat{F}}_{ST},H_{0}\right)}{n}\phi \left(c\right)+o_{p}(\frac{1}{n}), \end{array}$$ (19)

where ${\hat{F}}_{ST}$ is defined through the marginal distribution ${\hat{F}}_{X}$ and ${\hat{F}}_{Y}$ and the transformation at Step 5 in our algorithm and *ϕ*(*c*) and Φ(⋅) are the standard normal density and cumulative distribution functions, respectively. Eqs (18) and (19) yields the expression for the Kolmogorov-Smirnov distance as

$$\begin{array}{ccc} & & \underset{c}{\text{sup}}|P(\frac{{\hat{\rho }}_{0}\left(S,T\right)-\rho_{0}}{s_{{\hat{\rho }}_{0}}\left(S,T\right)}\leq c|F_{ST},H_{0})-P(\frac{{\hat{\rho }}_{0}^{*}\left(S,T\right)-\rho_{0}}{s_{{\hat{\rho }}_{0}^{*}\left(S,T\right)}}\leq c|{\hat{F}}_{ST}^{*},H_{0})|,\\ & = & \underset{c}{\text{sup}}|\frac{p_{1}\left(c|F_{ST},H_{0}\right)-p_{1}\left(c|{\hat{F}}_{ST},H_{0}\right)}{\sqrt{n}}+\frac{p_{2}\left(c|F_{ST},H_{0}\right)-p_{2}\left(c|{\hat{F}}_{ST}^{*},H_{0}\right)}{n}|\phi \left(c\right)\\ & + & o_{P}(\frac{1}{n}), \end{array}$$ (20)

where the polynomials *p*_*i*_ depend on *F*_*ST*_ in a smooth way. If the distance between between ${\hat{F}}_{ST}-F_{ST}$ is $O_{P}\left(\sqrt{n}\right)$ then expression (20) holds. Again, recall that ${\hat{F}}_{ST}$ is defined through the marginal distribution ${\hat{F}}_{X}$ and ${\hat{F}}_{Y}$ and the transformation at Step 5 in our algorithm, where using standard techniques it is straightforward to show ${\hat{F}}_{X}-F_{X}=O_{P}\left(\sqrt{n}\right)$ and ${\hat{F}}_{Y}-F_{Y}=O_{P}\left(\sqrt{n}\right)$ and that $\bar{X}\to \mu_{X}$, *s*_*X*_ → *σ*_*X*_, $\bar{Y}\to \mu_{Y}$ and *s*_*Y*_ → *σ*_*Y*_, where *μ*_*X*_, *σ*_*X*_, *μ*_*Y*_ and *σ*_*Y*_ are constants. In other words, the distribution of $\frac{{\hat{\rho }}_{0}^{*}\left(S,T\right)-\rho_{0}}{s_{{\hat{\rho }}_{0}^{*}\left(S,T\right)}}\to AN\left(0,1\right)$ under the assumptions provided and consistent with the original distribution of $\frac{{\hat{\rho }}_{0}\left(X,Y\right)-\rho_{0}}{s_{{\hat{\rho }}_{0}\left(X,Y\right)}}$ as per Theorem 2.1.

**Comment 2.3**. For the special and important case *H*_0_: *ρ* = 0 the only practical difference between our approach and the permutation based method of DiCiccio and Romano [16] is that $s_{{\hat{\rho }}_{0}^{*}\left(S,T\right)}$ is recalculated per each bootstrap replicate due to sampling with replacement mechanism whereas the permutation value is fixed across all permutations under the null. We do show however in our simulation study that the bootstrap test for *H*_0_: *ρ* = 0 does appear to have some cases where it works slightly better than the permutation test, likely due to a finer grid of values in the resampling scheme of with replacement as compared to without replacement.

## Results

For our simulation study we tested *H*_0_: *ρ* = *ρ*_0_ versus *H*_1_: *ρ* > *ρ*_0_ for *ρ*_0_ = 0.0, 0.3 and 0.6 with sample sizes *n* = 10, 25, 50, 100, 200. Each simulation utilized 10,000 Monte Carlo replications and the bootstrap resampling algorithm used *B* = 500 resamples. We compared Type I error control between the studentized permutation test of DiCiccio and Romano [16], the new bootstrap test, Fisher’s z-transform and the straight large sample approximation given Eq (5) using sample moment estimators for the variance estimation. The tests examined the Type I error control *α* = 0.05 as was performed by DiCiccio and Romano [16].

We utilized the same marginal distributions for *F*_*X*_ and *F*_*Y*_ from DiCiccio and Romano [16] page 1218 for the case *H*_0_: *ρ* = 0 for our simulation study:

1. Multivariate normal (MVN) with mean zero and identity covariance.
2. Multivariate t-distribution (Multivariate *t*_5_) with 5 degrees of freedom.
3. Exponential given as (*X*, *Y*) = *rS*′*u* where $S=diag(\sqrt{2},1)$, *u* is uniformaly distributed on the two dimensional unit circle.
4. Circular given as the uniform distribution on a two dimensional unit circle.
5. *t*_4.1_ where *X* = *W* + *Z* and *Y* = *W* − *Z*, where *W* and *Z* are i.i.d. *t*_4.1_ random variables.

One can see DiCiccio and Romano [16] for more detail regarding these specific distributions.

For the general case *H*_0_: *ρ* = *ρ*_0_ we utilized the marginal distributions above, standardized the marginal variables *X* and *Y* to mean = 0 and variance = 1 and applied the transformation at (11) under *H*_0_. The results are contained in Tables 1–3.

**Table 1:** Rejection Probabilities at *α* = 0.05 for bootstrap, asymptotic and Fisher’s z tests for *H*_*o*_: *ρ* = *ρ*_0_ versus *H*_1_: *ρ* > *ρ*_0_ using the sample correlation coefficient.

Distribution	*ρ*_0_	Test	*n*				
			10	25	50	100	200
MVN	0.0	Bootstrap	0.0533	0.0514	0.0493	0.0464	0.0516
		Asymptotic	0.1011	0.0698	0.0586	0.0523	0.0543
		Fisher’s z	0.0497	0.0513	0.0510	0.0482	0.0519
		Stud Perm*	0.0470	0.0525	0.0525	0.0511	0.0561
	0.3	Bootstrap	0.0571	0.0538	0.0526	0.0533	0.0496
		Asymptotic	0.1076	0.0687	0.0599	0.0551	0.0511
		Fisher’s z	0.0492	0.0477	0.0491	0.0493	0.0483
	0.6	Bootstrap	0.0641	0.0619	0.0549	0.0571	0.0543
		Asymptotic	0.1136	0.0752	0.0601	0.0591	0.0545
		Fisher’s z	0.0492	0.0474	0.0486	0.0509	0.0494
Exponential	0.0	Bootstrap	0.0524	0.0461	0.0511	0.0474	0.0504
		Asymptotic	0.0959	0.0643	0.0603	0.0512	0.0525
		Fisher’s z	0.0279	0.0214	0.0237	0.0231	0.0234
		Stud Perm*	0.0679	0.0508	0.0476	0.0502	0.0485
	0.3	Bootstrap	0.0599	0.0545	0.0515	0.0560	0.0542
		Asymptotic	0.1070	0.0725	0.0599	0.0597	0.0565
		Fisher’s z	0.0272	0.0245	0.0223	0.0236	0.0243
	0.6	Bootstrap	0.0603	0.0604	0.0570	0.0558	0.0529
		Asymptotic	0.1072	0.0761	0.0627	0.0591	0.0533
		Fisher’s z	0.0244	0.0250	0.0222	0.0240	0.0213

* Numbers abstracted from DiCiccio and Romano (2017)

**Table 2:** Rejection Probabilities at *α* = 0.05 for bootstrap, asymptotic and Fisher’s z tests for *H*_*o*_: *ρ* = *ρ*_0_ versus *H*_1_: *ρ* > *ρ*_0_ using the sample correlation coefficient.

Distribution	*ρ*_0_	Test	*n*				
			10	25	50	100	200
*t*_4.1_	0.0	Bootstrap	0.0485	0.0432	0.0450	0.0428	0.0451
		Asymptotic	0.0991	0.0608	0.0550	0.0467	0.0462
		Fisher’s z	0.1167	0.1438	0.1657	0.1769	0.1900
		Stud Perm*	0.0444	0.0428	0.0426	0.0442	0.0391
	0.3	Bootstrap	0.0533	0.0546	0.0512	0.0527	0.0527
		Asymptotic	0.1006	0.0638	0.0556	0.0516	0.0494
		Fisher’s z	0.1036	0.1371	0.1604	0.1767	0.1847
	0.6	Bootstrap	0.0576	0.0570	0.0587	0.0659	0.0570
		Asymptotic	0.1071	0.0621	0.0552	0.0580	0.0469
		Fisher’s z	0.0991	0.1187	0.1471	0.1591	0.1619
Multivariate *t*_5_	0.0	Bootstrap	0.0473	0.0477	0.0470	0.0523	0.0521
		Asymptotic	0.0947	0.0638	0.0545	0.0543	0.0543
		Fisher’s z	0.0497	0.0466	0.0471	0.0513	0.0516
		Stud Perm*	0.0507	0.0462	0.0460	0.0456	0.0471
	0.3	Bootstrap	0.0538	0.0530	0.0475	0.0440	0.0479
		Asymptotic	0.1084	0.0689	0.0579	0.0505	0.0510
		Fisher’s z	0.0593	0.0612	0.0609	0.0636	0.0661
	0.6	Bootstrap	0.0516	0.0509	0.0459	0.0407	0.0412
		Asymptotic	0.1069	0.0687	0.0570	0.0493	0.0489
		Fisher’s z	0.0763	0.0874	0.0878	0.0981	0.1027

*Numbers abstracted from DiCiccio and Romano (2017)

**Table 3:** Rejection Probabilities at *α* = 0.05 for bootstrap, asymptotic and Fisher’s z tests for *H*_*o*_: *ρ* = *ρ*_0_ versus *H*_1_: *ρ* > *ρ*_0_ using the sample correlation coefficient.

Distribution	*ρ*_0_	Test	*n*				
			10	25	50	100	200
Circular	0.0	Bootstrap	0.0570	0.0480	0.0514	0.0540	0.0438
		Asymptotic	0.1010	0.0645	0.0605	0.0570	0.0455
		Fisher’s z	0.0279	0.0240	0.0242	0.0234	0.0193
		Stud Perm*	0.0674	0.0468	0.0488	0.0484	0.0521
	0.3	Bootstrap	0.0612	0.0551	0.0511	0.0535	0.0503
		Asymptotic	0.1039	0.0701	0.0603	0.0552	0.0521
		Fisher’s z	0.0273	0.0231	0.0233	0.0198	0.0211
	0.6	Bootstrap	0.0622	0.0595	0.0581	0.0533	0.0544
		Asymptotic	0.1097	0.0751	0.0643	0.0568	0.0550
		Fisher’s z	0.0254	0.0227	0.0243	0.0224	0.0227

* Numbers abstracted from DiCiccio and Romano (2017)

The first striking point to make is how inflated the Type I error can be for the test based on Fisher’s z-transformation. For the *t*_4.1_ at *H*_0_: *ρ* = 0.3 we see an estimated Type I error rate of 0.1847 at *n* − 200. In the other direction the test based on Fisher’s z-transformation can also have much lower than anticipated Type error rate, e.g. see *H*_0_: *ρ* = 0.3, *n* = 100 for the circular distribution.

In terms of the straight large sample approximation [6] we see that it performs fairly well across all distributions for samples of size *n* ≥ 25, but has inflated Type I error rates for *n* = 10 usually twice the desired *α*.

For the specific case *H*_0_: *ρ*_0_ = 0 we see that the bootstrap test and permutation test are comparable with the bootstrap test actually subtlety outperforming the permutation test, even for large sample sizes, for certain scenarios. The bootstrap test and asymptotic tests also have similar properties for large sample sizes thus heuristically validating our theoretical arguments from the previous section. The bootstrap test appears to work well across all scenarios in terms of Type I error control and thusly would appear to be a robust method for testing the general hypothesis *H*_0_: *ρ* = *ρ*_0_.

In Table 4 we compared the power of the bootstrap test versus the exact Student’s t-test under multivariate normality assumptions. We see that the bootstrap test compares favorably to a test based on optimal assumptions and one that is limited by the form of the null hypothesis, i.e. the exact Student t-test for testing *H*_0_: *ρ*_0_ = 0 is specific to this form of the null hypothesis and does not generalize to other values of *ρ*_0_. We see from Table 4 that the exact test has a slight power advantage for small samples (*n* = 10), which dimensions rapidly as a function of larger sample sizes. A similar analogy would be found comparing the Wilcoxon rank-sum test versus the two-sample t-test in terms of efficiency where the two-sample t-test is only more efficient in general to the Wilcoxon rank-sum test under the strict and often unrealistic assumpion of normality.

**Table 4:** Power comparisons at *α* = 0.05 for bootstrap versus exact Student’s t-test for *H*_*o*_: *ρ* = 0 versus *H*_1_: *ρ* > 0 using the sample correlation coefficient.

Distribution	*ρ*	Test	*n*		
			10	25	50
MVN	0.3	Bootstrap	0.186	0.402	0.670
		t-test	0.201	0.415	0.677
	0.6	Bootstrap	0.484	0.917	0.999
		t-test	0.562	0.940	0.999

## Example

**Fig 1:**
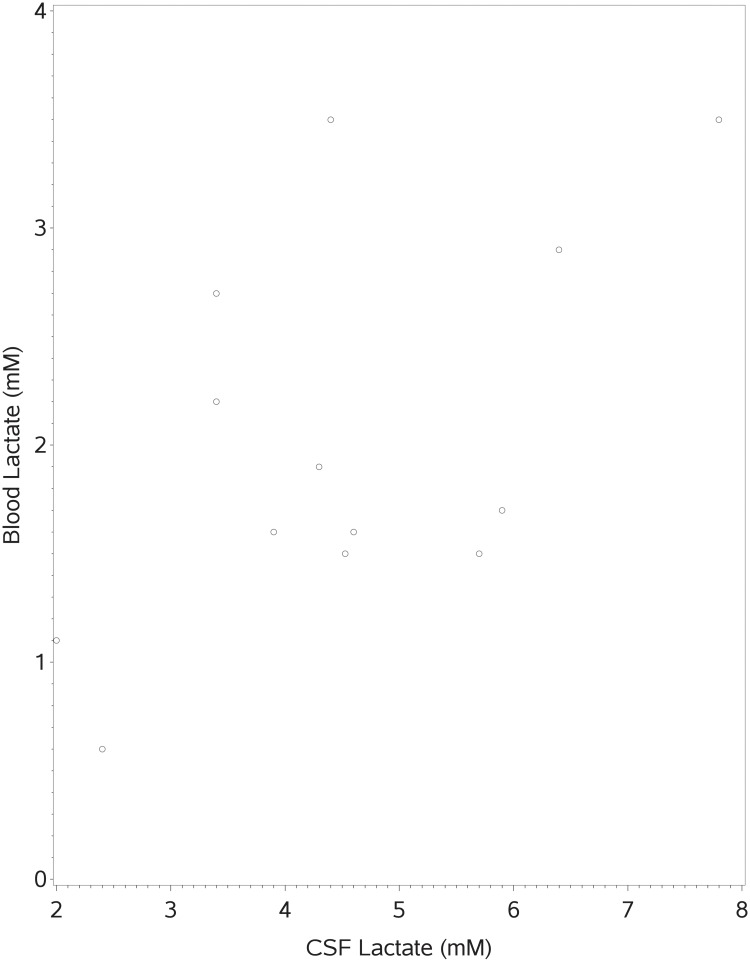
Scatterplot of blood lactate levels versus CSF lactate levels.

As a straightforward example of our approach we tested *H*_0_: *ρ* = *ρ*_0_ versus *H*_1_: *ρ* > *ρ*_0_ for *ρ*_0_ = 0 and *ρ*_0_ = 0.3 using lactate levels measured in the blood and the cerebrospinal fluid (CSF) in 13 female subjects [20]. The data are provided in Table 5. A scatterplot of the paired data is given in Fig 1.

**Table 5:** Example blood and CSF lactate levels on *n* = 13 female subjects.

	Blood	CSF
Subject	Lactate (mM)	Lactate (mM)
1	3.5	7.800
2	2.7	3.400
3	1.7	5.900
4	2.9	6.400
5	0.6	2.400
6	1.1	2.000
7	3.5	4.400
8	1.9	4.300
9	1.5	5.700
10	1.6	3.900
11	2.2	3.400
12	1.5	4.528
13	1.6	4.600

We used SAS 9.4 (SAS Institute Cary, NC) to test the marginal normality of the data using the Shapiro-Wilk test, the multivariate skewness and kurtosis coming from a bivariate normal distribution using the built in Mardia tests and the overall test of bivariate normality using the Henze-Zirkler T test. The results from SAS given in Table 6. We see that none of the tests rejects a given aspect of normality at *α* = 0.05. We do note that there is low power to do so given *n* = 13.

**Table 6:** Results of normality tests for example data.

Normality Test			
Equation	Test Statistic	Value	Prob
blood	Shapiro-Wilk W	0.93	0.3457
csf	Shapiro-Wilk W	0.97	0.9115
	Mardia Skewness	1.81	0.7698
	Mardia Kurtosis	-0.77	0.4386
	Henze-Zirkler T	0.35	0.4613

The estimated correlation between blood and CSF lactate levels was $\hat{\rho }=0.57$ For the test *H*_0_: *ρ* = 0 versus *H*_1_: *ρ* > 0 the p-values were 0.0198, 0.0398, 0.0770 and 0.0628 for the Fisher’s z transformation based test, the large sample test, the bootstrap test and the studentized permutation test of DiCiccio and Romano [16], respectively. The bootstrap and permutation tests used 5,000 resamples. If were testing at level *α* = 0.05 there is agreement between the permutation and bootstrap test of not rejecting *H*_0_ while the test based on Fisher’s z transformation and the large sample approximation indicate to reject *H*_0_. Given the small sample size and the results of our simulation study we would recommend that the permutation or bootstrap p-values are likely the more useful calculations in terms of drawing the correct conclusion, particularly given the closeness of the values between the Fisher’s z transformation based p-value and the large sample approximation, which we demonstrated did not control the Type I error in small samples.

For the test *H*_0_: *ρ* = 0.3 versus *H*_1_: *ρ* > 0.3 the p-values were 0.149, 0.1399, 0.1792 and not applicable (doesn’t generalize) for the Fisher’s z based test, the large sample test, the bootstrap test and the studentized permutation test of DiCiccio and Romano [16], respectively. All three tests do not reject *H*_0_ at *α* = 0.05.

## Discussion and conclusion

In this note we present a robust bootstrap test with good Type I error control for testing the general hypothesis *H*_0_: *ρ* = *ρ*_0_. The test was inspired by the studentized permutation given by DiCiccio and Romano [16] for testing *H*_0_: *ρ* = 0, which was proven to be exact in certain scenarios and asymptotically correct overall. We believe that statistical software packages should employ both the bootstrap test and the studentized permutation given by DiCiccio and Romano [16] as the default tests over tests based on bivariate normal assumptions. In addition, it should be noted that the bootstrap test can be inverted to form a confidence interval about *ρ*. This will be examined in our future work.
